# Evaluation of a hybrid battery digital twin for joint SOC–SOE estimation under real driving conditions

**DOI:** 10.3389/frai.2026.1816237

**Published:** 2026-09-14

**Authors:** Sushil Krishnan, Yokesh Babu Sundaresan

**Affiliations:** School of Computer Science and Engineering, Vellore Institute of Technology, Vellore, Tamil Nadu, India

**Keywords:** battery state estimation, digital twin, electric mobility, hybrid modeling, real driving conditions

## Abstract

Reliable estimation of battery state of charge (SOC) and state of energy (SOE) under real world dynamic driving conditions is essential for battery management systems. However, traditional physics-based models and purely data-driven models struggle to adapt and generalize. This work proposes Closed-Loop Hybrid Digital Twin (CL-HDT) that synergizes adaptive physics twin, recurrent residual learning, physics guided optimization, and recursive state feedback for joint SOC-SOE estimation. Adaptive physics twin generates physically consistent reference estimate and residual from closed-loop recurrent model recursively corrects it. The proposed CL-HDT was developed with a dataset containing 30 real-world electric cycle driving trips, split with leakage-safe strategy into training, validation, and locked holdout test set. To analyze generalizability, leave-one-trip-out cross-validation (LOTO-CV) was applied within training set. The proposed architecture was benchmarked against five baselines including Extended Kalman Filter (EKF), Gated Recurrent Unit (GRU), Feature-Fusion Recurrent estimator (FFR), Residual-Guided Hybrid estimator (RGH), and Adaptive Physics Twin (APT). All the baselines and CL-HDT were trained and evaluated under identical pipeline to ensure fair comparison. CL-HDT attained strongest generalization in LOTO-CV with mean RMSE values of 0.7078% and 0.6883% for SOC and SOE respectively, while maintaining competitive performance in locked holdout test. Apart from aggregate metrics, CL-HDT was further evaluated using residual correction analysis, tracking curves, drift curves, component-wise ablation, error distribution, and ambient condition analysis to demonstrate its robustness and ability to mitigate accumulation of error over longer real-world trips. This approach offers a physically consistent solution for next-generation battery management systems under realistic driving conditions to support safety and battery life.

## 1 Introduction

Electric mobility transition demands real time battery monitoring systems, that will accurately work across various operating conditions. These systems usually track state of charge (SOC) and state of energy (SOE). While SOC indicates range, charging control and safety constraints, SOE measures usable energy under varying load and operating conditions (Mišljenović et al., 2026). Estimation error in either of the measure adversely impacts safety, energy efficiency, and user trust. Lightweight electrics vehicles like electric cycles face the impacts of these challenges to greater extent, as they have limited battery capacity and operate in dynamic environment (Reddy et al., 2025). Improved SOC-SOE joint estimation can progress sustainability as it supports battery health, minimizes premature maintenance, and makes electric mobility resource efficient.

Real driving conditions are often complex with rapid acceleration/deceleration, regenerative events, interruptions, and load variations (Chen et al., 2025). Most studies on battery state estimation are carried out with datasets generated in controlled setup; such estimators greatly struggle to generalize in real world scenarios as they interpolate within trip profiles. Along with modeling approaches, strong evaluation system on real world dataset is essential to observe actual deployment behavior and generalizability of estimators.

Classical physics-based estimators like Extended Kalman Filter based equivalent circuit models remain relevant as they offer explainable and computationally optimal predictions. However, their performance is bound to data fidelity and varying behavior can downgrade them. Pure neural estimators learn complex nonlinear trends, but they are influenced by training distributions and can produce physically inconsistent predictions. To exploit strengths of these both approaches, hybrid models can be used to learn residuals and also estimate physically consistent states (Zhao et al., 2025).

In this work, the proposed Closed-Loop Hybrid Digital Twin (CL-HDT) for SOC-SOE joint estimation was developed and evaluated on a real-world e-cycle data with strong evaluation strategy. The proposed framework synergizes an adaptive physics twin and a recurrent residual correction network under closed-loop feedback to maintain physically consistent estimates and progressively improve performance. Against conventional hybrid estimator's single-pass residual correction, CL-HDT recursively updates adaptive physics model with corrected SOC and SOE to adapt in dynamic conditions and maintain estimation stability over long trips. To demonstrate the effectiveness of proposed architecture, it was benchmarked against five varying baselines spanning physics based, data driven and hybrid approaches. The baselines included Extended Kalman Filter (EKF), Gated Recurrent Unit (GRU), Feature-Fusion Recurrent estimator (FFR), Residual-Guided Hybrid estimator (RGH), and Adaptive Physics Twin (APT). Proposed model and all baselines were evaluated in an identical leakage-safe framework that involved strict trip-wise partitioning, leave-one-trip-out cross validation (LOTO-CV), and a locked holdout test set. Hyperparameter tuning for configurable models were performed exclusively with training data and structured sweep to avoid data leakage and ensure reproducibility.

The contributions of this paper are:

- Closed-Loop Hybrid Digital Twin (CL-HDT) is proposed for SOC-SOE co-estimation by synergizing adaptive physics twin, recurrent residual learning, and closed-loop recursive feedback.
- Rigorous reproducible experimental framework is highlighted, which included strict trip-wise data partitioning, leakage-safe preprocessing, LOTO-CV, transparent hyperparameter tuning, and locked holdout test set to ensure generalizability and unbiased evaluation.
- Proposed CL-HDT is benchmarked against wide variety of baselines in identical experimental setup to fairly observe the superiority of proposed approach in real world data.
- Behavior of proposed CL-HDT is comprehensively analyzed through ablation, residual analysis, tracking curve, drift analysis, error distribution, and ambient condition analysis to observe the robustness of proposed framework.

The article is organized as follows. Section 2 provides insights on existing literature in SOC/SOE estimation and digital twins. Section 3 describes complete methodology from dataset description to evaluation system. Section 4 discusses all the experimental results. Section 5 concludes the article and highlights future work.

## 2 Literature review

Classical battery state estimation models are built with equivalent circuit models (ECMs) like Thevenin or higher-order RC networks, they have been demonstrated to model lithium-ion voltage dynamics (Shan et al., 2025). Often these models are combined with recursive filters like EKF and Unscented Kalman Filter to enable noise safe online updates for SOC or joint SOC-SOE estimation (Yu and Yao, 2025). H-infinity filters and sliding mode observers (SMO) have been observed to improve robustness against disturbances and also model uncertainty, however SMO can suffer with chattering at higher frequencies (Gaufan et al., 2025). Classical models are apt for onboard battery management as they are computationally inexpensive, physically consistent and embedded compatible (Ahmed et al., 2025). Their adoption is limited in dynamic scenarios as they are strongly parameter dependent. Adaptive re-identification is required to maintain performance (Isa et al., 2025).

Data driven estimation of SOC utilizing neural models bypass electrochemical relations by directly learning nonlinear mapping from measurable variables like voltage, current and temperature to SOC (Yilmaz et al., 2025). Recurrent models like LSTM are of great interest as gated memory maintains performance over long temporal modeling (Ahn et al., 2025). In deployment perspective, GRU based models with fewer parameter and competitive accuracy are preferred (Sharma et al., 2026). While CNN-RNN hybrids perform by combining local feature extraction with sequence learning, Transformer models model long range trends using self-attention (Dini and Paolini, 2025). These data intensive approaches lose robustness on unseen or extreme conditions outside training distribution (Wang et al., 2025a), and also remains as “black boxes” that lack physical consistency observed in physics-based models.

SOE estimation remains sparsely explored than SOC despite being recognized as vital indicator for energy management (Alamin et al., 2025). While SOC represent charge-fraction, SOE directly indicates usable energy. Joint estimation of SOC-SOE is being favored to exploit the interdependency between them to reduce errors and avoid redundancy of multiple estimators (Zou et al., 2025). Recent works have focused on co-estimation that include SOC, SOE, and other power related targets using methods like meta-learning with square-root UKF (Wang et al., 2025b). However, these advancements have not addressed the gaps in robustness at extreme variations in temperature, load, and parameters (Shrivastava et al., 2022).

Hybrid methods combining physical structures and adaptive learning are found to fill the gaps of their pure counterparts. Commonly residual learning is used, where a physics branch like discrete-time ECM provides baseline trajectory, which is corrected by a neural branch that learns mismatch (Apribowo et al., 2025). Physics-informed neural approaches directly constraint optimization to improve physical consistency under sparse sensing (Jin et al., 2025). Internal degradation such as SEI-based evolutions are also observed to be modeled by deep physics coupled architectures (Tian et al., 2025). These methods are challenged by their computational burden and delayed convergence due to sensitivity to balance between data-fit and physics-fit (Yang et al., 2025).

Digital twin (DT) further improves the battery management by maintaining a continuously updated virtual battery representation supported by telemetry and physics consistent models (Farooq et al., 2026). DT architectures are usually hierarchical involving layers like sensing and edge to cloud simulation (Qasem et al., 2026). Hybrid DT fuse mechanistic battery model for interpretability and data intensive models for predictive performance (Najafi-Shad et al., 2026). Defining upside is feedback, where online updates are done on model state and parameters under varying operating conditions like load, environment, and aging (Ramesh Babu et al., 2025). SOC and SOE remain prime operational indicators for safety, range, and power management (Wu et al., 2024). Recent works have explored practical challenges such as estimation on partially discharged state and limited initial data (Jafari and Byun, 2022).

Adoption of models developed in controlled conditions for real driving is challenging, even standardized cycles (e.g., HWFET, US06) lack highly non-stationary behavior of irregular transients and current spikes (Pande et al., 2025). Distribution shift arising from charge-discharge cycles and condition variations weaken reliability of model. To avoid data leakage and bias, strictly disjoint and independent evaluation sets are required (Li et al., 2025). Robustness of model is essential across sensor noise and temperature variations (Fouka et al., 2025), chemical and load heterogeneity (Haraz et al., 2025), and congested operating environments (Miao et al., 2025).

Robust joint estimation of SOC-SOE under real world scenarios remains challenging despite significant developments in physics-based modeling, data-driven modeling, and hybrid modeling. Existing methods struggle to continuously adapt to evolving dynamics and maintain physical consistency over long trips. This works proposes CL-HDT to address the gaps by validating it under strong leakage safe trip wise evaluation and comprehensive analysis. Improved estimation can support safer electric mobility, enhance range confidence, and minimize environmental footprint by reducing unnecessary wear and tear. Thereby leaving a positive impact on sustainability.

## 3 Methodology

The workflow adopted for developing and evaluating the proposed CL-HDT is illustrated in Figure 1. Leakage safe trip level split is applied on raw trip data to obtain train, validation, and test splits. Additionally, LOTO-CV is applied within train split trips. Fold specific preprocessing for temporal alignment, feature engineering, and windowing is performed. While train and validation sets are used for model training and hyperparameter tuning, test set is exclusively held out for evaluation. To ensure unbiased analysis, all baselines and proposed model are benchmarked under identical workflow. Further analysis for deep observations like ablation and drift analysis were performed on proposed model.

**Figure 1:**
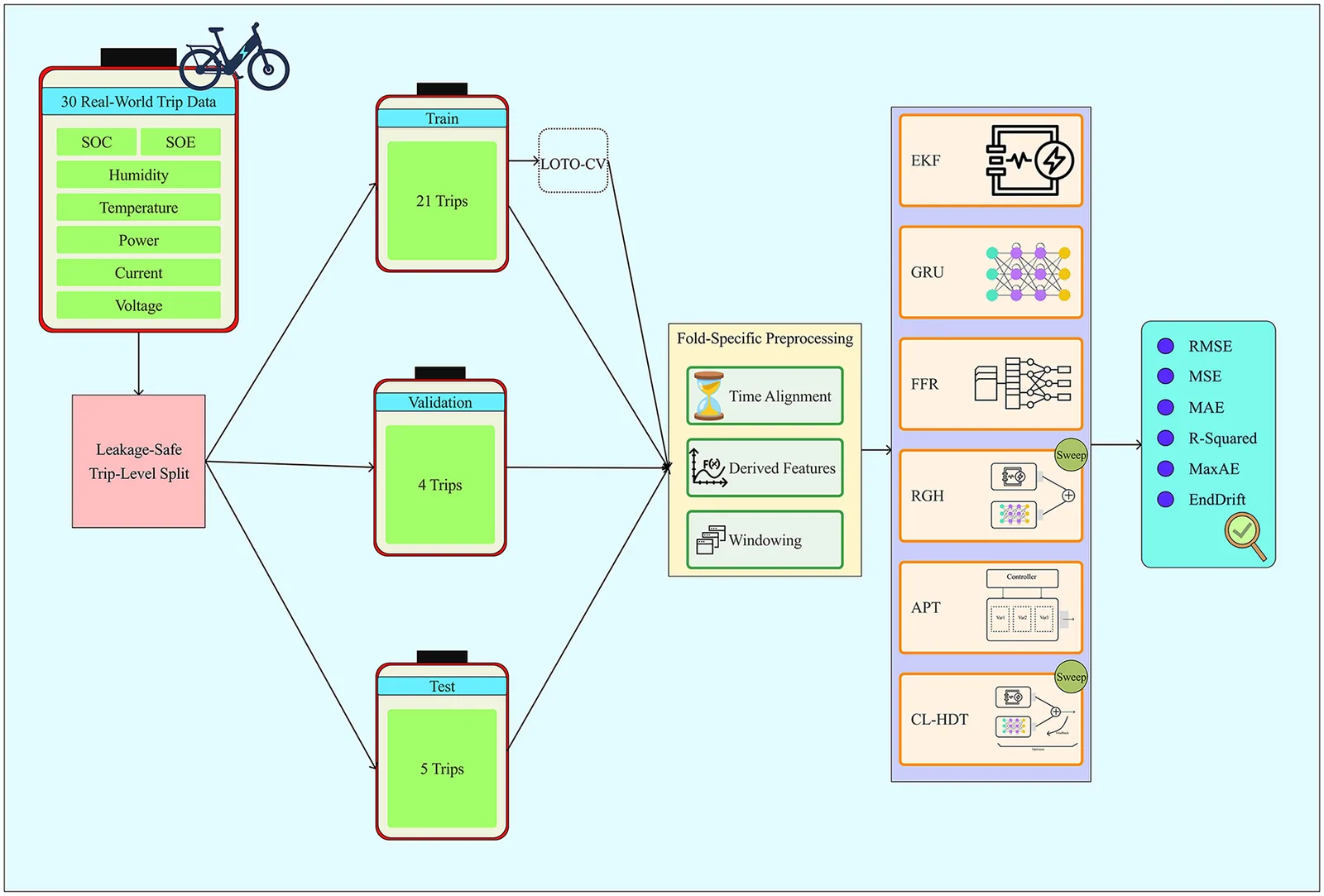
Workflow architecture adopted in developing proposed CL-HDT for SOC-SOE joint estimation.

### 3.1 Dataset description

This work uses lithium-ion battery data collected from electric cycles operated on Indian highways. It includes 30 trips with real world variations and nine measurement columns are present in each trip data. The columns include Time, Volts, Amps, Watts, Temp, Humid, Elapsed_ms, SOC_percent, and SOE_percent. Volts and Amps represent electrical measurements, Watts represent instantaneous power, and Temp and Humid represent ambient conditions. SOC_percent and SOE_percent is taken as targets. Each trip is a continuous session and considered as independent data record. The trip level processing helps in representing realistic driving behavior and supports joint estimation in real world conditions (Manivannan et al., 2026).

### 3.2 Experimental design

To avoid data leakage and ensure realistic evaluation, splitting is carried out at trip level. The 30 trips were split in 70:15:15 ratio to yield mutually exclusive subsets of 21, 4, and 5 trips for training, validation, and locked holdout test, respectively. LOTO-CV is performed within training set to observe cross trip generalization. Final evaluation with test set is carried out with model trained with train and validation sets.

Data dependent operations like feature scaling and parameter calibration were restricted within folds or appropriate splits to prevent leakage and optimistic bias. Post hyperparameter tuning with LOTO-CV, final configuration is trained using train data and validation data and then evaluated with unseen test data. This design assures conservative deployment performance observation and fair comparison of all models.

### 3.3 Preprocessing and feature engineering

Following data splitting, preprocessing and feature engineering are carried out within each fold. Elapsed_ms is transformed into seconds and sampling interval Δt is calculated for each observation. To maintain sequence consistency, non-positive and inconsistent increments are replaced with median valid interval of respective trip. Final features included both original measurements and derived features from Volts, Amps, Watts, Temp, and Humid to capture temporal trends (Harinarayanan and Balamurugan, 2025). It includes first-order derivative of voltage and current as defined in Equations 1 and 2, respectively. Cumulative charge feature represents integration of current flow as calculated in Equation 3.


$$\begin{array}{l} \frac{dV}{dt}=\frac{V_{t}-V_{t-1}}{\Delta t} \end{array}$$ (1)

$$\begin{array}{l} \frac{dI}{dt}=\frac{I_{t}-I_{t-1}}{\Delta t} \end{array}$$ (2)

$$\begin{array}{l} Q_{int}\left(t\right)=\overset{t}{\underset{k=1}{\sum }}\frac{-I_{k}\Delta t_{k}}{3600} \end{array}$$ (3)

The additionally engineered featured support the model in learning transient responses and charge throughput to better predict SOC-SOE evolution. Neural and hybrid models handle sequential modeling through sliding windows of fixed size L. Equation 4 depicts a window ending at time t, where x_t_ is feature vector. Prediction corresponds to SOC and SOE percentages at final time step.


$$\begin{array}{l} X_{t}=\left[x_{t-L+1},x_{t-L+2},\ldots ,x_{t}\right] \end{array}$$ (4)

Standardization is used for feature scaling as represented in Equation 5, where statistical measures like mean and standard deviation are calculated exclusively within folds. Same params are applied for scaling validation and test sets without recalculation to ensure independence of evaluation and numerical stability during optimization.


$$x^{\prime }=\frac{x-\mu_{train}}{\sigma_{train}}$$ (5)

### 3.4 Baseline model formulation

#### 3.4.1 Extended Kalman Filter (EKF)

Classical baseline is implemented as a first-order Thevenin ECM with an EKF for recursive state estimation (Sangeetha et al., 2024). Equation 6 represents the state vector, where SOC_k_ is state of charge at time step k and V_p, k_ is RC branch polarization voltage.


$$\begin{array}{l} x_{k}=\left[\begin{array}{c} SOC_{k}\\ V_{p,k} \end{array}\right] \end{array}$$ (6)

SOC evolution follows coulomb counting as calculated in Equation 7, where I_k_ is measured current (positive for discharge), Δt_k_ is sampling interval in seconds, Q_n_ is the nominal battery capacity in ampere-hour and the factor 3,600 converts seconds to hours.


$$\begin{array}{l} SOC_{k}=SOC_{k-1}-\frac{I_{k}\Delta t_{k}}{3600Q_{n}} \end{array}$$ (7)

Polarization voltage and terminal voltages are modeled using Equations 8 and 9 respectively, where R_0_ denote ohmic resistance, R_1_ denote RC branch resistance, C_1_ denote RC branch capacitance, and OCV(.) represent open-circuit voltage-SOC relationship. OCV-SOC curve is approximated by a polynomial fitted on training trips.


$$\begin{array}{l} V_{p,\;k}=\;e^{\;-\frac{\Delta t_{k}}{\tau }\;}V_{p,k-1}+\;R_{1}\left(1-e^{\;-\frac{\Delta t_{k}}{\tau }\;}\right)I_{k},\tau =R_{1}C_{1} \end{array}$$ (8)

$$\begin{array}{l} {\hat{V}}_{k}=OCV\left(\;SOC_{k}\right)-I_{k}R_{0}-V_{p,k}\left(9\right) \end{array}$$ (9)

At each time step, EKF performs first-order linearization as model is nonlinear in SOC_k_. While state prediction and covariance propagation are modeled by standard EKF as in Equation 10, measure updates are represented by Equations 11–13, where F_k_ is Jacobian of state transition function, Q is process noise covariance matrix, H_k_ is Jacobian of measurement function, and R is measurement noise covariance.


$$\begin{array}{l} {\hat{x}}_{k|k-1}=f\left(x_{k-1},u_{k}\right),P_{k|k-1}=F_{k}P_{k-1}F_{k}^{T}+Q \end{array}$$ (10)

$$\begin{array}{l} K_{k}=P_{k|k-1}H_{k}^{T}{\left(H_{k}P_{k|k-1}H_{k}^{T}+R\right)}^{-1} \end{array}$$ (11)

$$\begin{array}{l} {\hat{x}}_{k}={\hat{x}}_{k|k-1}+K_{k}\left(V_{k}-{\hat{V}}_{k}\right) \end{array}$$ (12)

$$\begin{array}{l} P_{k}=\left(I-K_{k}H_{k}\right)P_{k|k-1} \end{array}$$ (13)

All the model parameters, including Q_n_, R_0_, R_1_, C_1_, and OCV coefficients are calibrated only with training trips of each fold. For final evaluation with locked test set, parameters are calibrated with complete train and validation set. Similarly, SOE is tracked with EKF state trajectory and evaluated. This approach offers a classical reference model against neural and hybrid approaches.

#### 3.4.2 Gated Recurrent Unit estimator (GRU)

Gated Recurrent Unit network is implemented for pure deep learning baseline. The network directly performs joint regression of SOC and SOE. Unlike EKF, it cannot enforce physical constraints and learns temporal dependencies instead. GRU hidden state update is defined by Equation 14 and for a given input window X_i_εR^LXF^, the sequential feature vector at time step t is denoted as x_t_.


$$\begin{array}{l} h_{t}=GRU\left(\;x_{t},\;h_{t-1}\right) \end{array}$$ (14)

Where h_t_ indicates the hidden state at time *t*. After processing a window of length L, final hidden state h_L_ is passed through a fully connected projection layer to get joint prediction as depicted in Equation 15, where the output is a vector containing predicted SOC and SOE for window endpoint, and W and b are learnable parameters. The model is trained with mean square error (MSE) loss over both targets as computed by Equation 16.


$$\begin{array}{l} {ŷ}_{i}=Wh_{L}+b \end{array}$$ (15)

$$\begin{array}{l} L_{data}=\frac{1}{N}\overset{N}{\underset{i=1}{\sum }}||{ŷ}_{i}-y_{i}|{|}_{2}^{2} \end{array}$$ (16)

The model is intentionally implemented in a compact way to maintain deployment feasibility. GRU is used as primarily data driven reference against EKF and hybrids (Tang et al., 2023).

#### 3.4.3 Feature-Fusion Recurrent estimator (FFR)

Feature-Fusion Recurrent estimator injects physics derived data into GRU input stream to act as a hybrid baseline. SOC predicted with EKF is appended as additional feature at each time step to examine the impact of feature level physics fusion without any feedback or residual coupling (Fan et al., 2025). Equation 17 demonstrates the augmented feature vector, where ${\text{SOC}}_{\text{t}}^{\text{EKF}}$ denote EKF's SOC prediction.


$$x_{t}^{'}=[x_{t},SOC_{t}^{EKF}]$$ (17)

Window level augmented input sequence is shown in Equation 18. While input dimensionality increased, recurrent update, output projection, and loss remain same as GRU. To preserve leakage-safe evaluation, EKF predictions are generated with EKF calibrations derived from training set alone. FFR is used to isolate the impact of physics guided feature augmentation and is an intermediate between pure data driven approach and structured hybrid/twin models.


$$X_{i}^{'}=[{x^{\prime }}_{i,1},{x^{\prime }}_{i,2},\ldots ,{x^{\prime }}_{i,L}]\;X_{i}^{'}\in {\mathbb{R}}^{L\;\times \;(F+1)}$$ (18)

#### 3.4.4 Residual-Guided Hybrid estimator (RGH)

Residual-Guided Hybrid estimator introduces structured residual correction by fusing physics branch and neural correction branch. While FFR performs feature level fusion, RGH corrects physics trajectory at output level (Shi et al., 2026). ECM based estimates from physics branch is depicted by Equation 19 and estimated correction for respective window from GRU based residual estimator is shown in Equation 20.


$$\begin{array}{l} y_{t}^{phys}=\left[SOC_{t}^{phys},SOE_{t}^{phys}\right] \end{array}$$ (19)

$$\begin{array}{l} \Delta y_{t}=\left[\Delta SOC_{t},\;\Delta SOE_{t}\right] \end{array}$$ (20)

Final prediction is sum of physics estimate and correction as calculated in Equation 21. Training uses a composite objective function represented by Equation 22, where L_data_ is combined SOC-SOE MSE and L_phys_ is soft regularizer for physical consistency. Regularization weight λ_phys_ is obtained though sweep on training folds. This architecture forces neural branch to stay consistent with physics estimate rather than learn from scratch and remains as a structured hybrid baseline between simple fusion and closed loop twins.


$$\begin{array}{l} {ŷ}_{t}=y_{t}^{phys}+\Delta y_{t} \end{array}$$ (21)

$$\begin{array}{l} L_{total}=L_{data}+\lambda_{phys}L_{phys} \end{array}$$ (22)

#### 3.4.5 Adaptive-Parameter Physics Twin (APT)

Adaptive-Parameter Physics Twin improves over fixed parameter ECM estimation by dynamically updating key physical quantities with varying operating conditions. Unlike RGH which also include neural residual correction, APT is physics only estimator with adaptive parameters (Lin et al., 2025). Equations 23 and 24 are used for SOC state update and resistance-related behavior modeling respectively, where ${\text{Q}}_{\text{n}}^{*}$ represents physically constrained adaptive capacity proxy and $\tau^{*}=R_{1}^{*}C_{1}^{*}$.


$$\begin{array}{l} SOC_{k}=SOC_{k-1}-\frac{I_{k}\Delta t_{k}}{\;3600Q_{n}^{*}} \end{array}$$ (23)

$$V_{p,k}=e^{-\frac{\Delta t_{k}}{\tau^{*}}}V_{p,(k-1)}+R_{1}^{*}(1-e^{-\frac{\Delta t_{k}}{\tau^{*}}})I_{k}$$ (24)

Similarly, terminal voltage also follows same format from EKF, but with adaptive terms. Parameter updates are conditioned based on operating conditions and constrained with predetermined bounds to avoid instability and ensure physical consistency. SOC and SOE are directly obtained from adaptive twin without residual correction and acts as adaptive physics benchmark before closed loop hybrid.

### 3.5 Proposed Closed-Loop Hybrid Digital Twin (CL-HDT)

The proposed Closed-Loop Hybrid Digital Twin is a unified battery state estimation framework, that improves upon APT by synergizing it with recurrent residual learning and recursive state feedback. Although APT dynamically adapts and RGH provide data driven residual correction, both lack mechanism to incorporate corrected states into subsequent predictions and remain open loop. This results in progressive accumulation of error over long driving sessions. CL-HDT overcomes this challenge by performing closed-loop estimation, where corrected states are recursively propagated through residual learning. The closed-loop mechanism along with hybrid approach ensures continuous refinement of SOC and SOE estimation and maintains physical consistency.

At every instance, APT provides initial estimation based on electrical measurements and adaptive ECM. The predicted quantities provide physically consistent reference trajectory for subsequent residual correction. Rather than directly predicting SOC and SOE, neural component only predicts the modeling error. This allows APT to preserve physical interpretability and neural component to compensate for nonlinear trends of real-world conditions that cannot be accounted by ECM.

The neural error estimation component captures temporal battery dynamics and is implemented as GRU cell. Unlike traditional recurrent estimators that consume current sequence alone, the proposed architecture additionally incorporates previously corrected state into recurrent update. This mechanism establishes explicit closed-loop feedback. Equation 25 represents hidden state update, where x_t_ denoting input vector and ŷ_t − 1_ denoting previous correction are concatenated and h_t_ denotes hidden state of recurrent network. By recursive incorporation of previous corrections, the network continuously adapts based on both current conditions and historical estimation behavior.


$$\begin{array}{l} h_{t}=GRUCell\left(\left[x_{t},\;{ŷ}_{t-1}\right],h_{t-1}\right) \end{array}$$ (25)

Further, corrections for both SOC and SOE are obtained by mapping recurrent representation to a joint residual through fully connected projection as represented in Equation 26. Where Δy_t_ is residual correction for APT predictions, it has both residuals.


$$\begin{array}{l} \Delta y_{t}=g\left(h_{t}\right) \end{array}$$ (26)

A learnable feedback coefficient shown in Equation 27 is introduced in CL-HDT to regulate recursive propagation. Where, θ is trainable parameter and σ(.) is sigmoid activation. The coefficient is bound as 0 ≤ α ≤ 1 and adaptively balances the contributions of current corrections and previous estimations to prevent abrupt changes and support sufficient adaptability. This feedback system differentiates CL-HDT from open loop hybrids.


$$\begin{array}{l} \alpha =\sigma \left(\theta \right) \end{array}$$ (27)

Final prediction is computed by adaptively summing APT predictions (${\text{y}}_{\text{t}}^{\text{phys}}$), learned residuals corrections (Δy_t_) and recursive feedback, as shown in Equation 28. While the first term corrects APT prediction using learned residual, second term recursively incorporates previous prediction. The proposed formulation preserves long term temporal stability and suppresses severe fluctuations caused by noise and short-term drifts.


$$\begin{array}{l} {ŷ}_{t}=\alpha \left(y_{t}^{phys}+\Delta y_{t}\right)+\left(1-\alpha \right){ŷ}_{t-1} \end{array}$$ (28)

Equation 29 defines the composite optimization of prediction accuracy and physical consistency, where L_data_ is joint MSE, L_charge_ enforces consistency between SOC trajectory and charge consumed according to Coulomb counting, L_anchor_ bounds corrections close to APT estimate, and L_dyn_ punish unrealistic deviations in trajectory with respect to APT. λ_phys_ regulate overall physics regularization, while β and γ regulate anchoring and dynamic consistency respectively. In this work λ_phys_ was fixed using hyperparameter tuning, and β and γ were kept at 0.05.


$$\begin{array}{l} L_{total}=L_{data}+\lambda_{phys}\left(L_{charge}+\beta {\text{L}}_{anchor}+\gamma L_{dyn}\right) \end{array}$$ (29)

The CL-HDT limits data driven modeling to residual prediction, thereby reducing its complexity and retaining physical interpretability of ECM. Further by bounding feedback coefficient to [0, 1] through sigmoid activation, stable transitions are enforced. Various physical constraints also discourage unrealistic state evolution. Synergistically, all these approaches promote stable estimation and mitigate accumulation of drift over long trips. The proposed framework therefore combines adaptive physical modeling, recurrent residual learning and closed-loop feedback for joint estimation of SOC and SOE. Against all the baselines CL-HDT is the only framework to exploit the advantages of all these approaches.

### 3.6 Training strategy and model selection

PyTorch was used to implement both neural and hybrid models. All the models were trained with an initial learning rate of 1 × 10^−3^, Adam optimizer, mini-batch gradient descent, and batch size of 256. Maximum epochs were set at 40 and early stopping was configured based on fold validation loss with a patience of 6 epochs and minimum threshold of 1 × 10^−4^. To ensure reproducibility, NumPy and PyTorch were set with fixed random seed of 42.

RGH and CL-HDT involved configurable components that required hyperparameter tuning. Complete 21-fold sweep was performed for RGH to determine physics loss and residual drift parameter, while a two-stage strategy involving initial 6-fold coarse search and final complete 21-fold sweep on top two configurations was done for CL-HDT. In both cases final model was retrained with parameters that gave lowest validation loss across all LOTO folds. The final training was done on complete training and validation set and then evaluated on unseen holdout test set with no post-test hyperparameter tuning to ensure fairness.

### 3.7 Evaluation metrics

Models are evaluated at trip level to ensure independence between driving scenarios. Metrics are individually reported for both SOC and SOE, where window level performances are mapped back to trip level metrics. Metrics are calculated as mean ± standard deviation for both 21 LOTO-CV folds and 5 locked holdout test trips separate tables.

The Equations 30–34 represents computed metrics mean absolute error (MAE), mean squared error (MSE), root mean squared error (RMSE), coefficient of determination (R^2^), and maximum absolute error (MaxAE), respectively (Kassa et al., 2025). Collectively they quantify prediction accuracy, prediction variance, goodness-of-fit, and worst-case error.


$$\begin{array}{l} MAE_{z}=\frac{1}{n}\overset{n}{\underset{i=1}{\sum }}|{ẑ}_{i}-z_{i}| \end{array}$$ (30)

$$MSE_{z}=\frac{1}{n}\sum_{i=1}^{n}{\left({\hat{z}}_{i}-z_{i}\right)}^{2}\;$$ (31)

$$\begin{array}{l} RMSE_{z}=\sqrt{MSE_{z}} \end{array}$$ (32)

$$\begin{array}{l} R_{z}^{2}=1-\frac{\sum_{i=1}^{n}{\left({ẑ}_{i}-z_{i}\right)}^{2}}{\overset{n}{\underset{i=1}{\sum }}{\left(z_{i}-\bar{z}\right)}^{2}} \end{array}$$ (33)

$$\begin{array}{l} MaxAE_{z}=\underset{i}{\max}|{ẑ}_{i}-z_{i}| \end{array}$$ (34)

Apart from standard performance metrics, to quantify accumulated bias over complete trip, end-of-trip drift (EndDrift) is calculated as in Equation 35. It is particularly relevant for battery management systems, where cumulative deviations at the end significantly impacts range and safety margins. Each model is compared using 12 metrics, which include RMSE, MSE, MAE, R^2^, MaxAE, and EndDrift for both SOC and SOE.


$$\begin{array}{l} Drift_{z}=|{ẑ}_{end}-z_{end}| \end{array}$$ (35)

## 4 Results and discussion

### 4.1 Hyperparameter tuning

Hyperparameter tuning was carried out using LOTO-CV prior to holdout evaluation to ensure fair assessment on unseen conditions. The complete 21-fold search of RGH resulted in λ_phys_ of 0.005 and Δ_cap_ of 40. The two-stage search of CL-HDT resulted in hidden dimension of 96, learning rate of 1 × 10^−3^ and λ_phys_ of 0.005.

### 4.2 Cross-trip generalization (LOTO-CV)

The cross-trip generalizability of models was evaluated using 21-fold leave-one-trip-out cross-validation. Their results for SOC and SOE are shown in Tables 1 and 2, respectively. Proposed CL-HDT is observed to deliver strongest performance for both the targets, achieving lowest mean RMSE, MSE, MAE, MaxAE, EndDrift, and highest R^2^. It shows substantial improvement (around 30% reduction in RMSE for both SOC and SOE) over strongest baseline RGH. It also has smaller standard deviation across metrics demonstrating robustness and generalizability across conditions. RGH being the strongest baseline, confirms residual guided learnings superior performance over pure data driven GRU and simple hybrid FFR. Success of CL-HDT further highlights the positive impacts of synergizing recursive state feedback and hybrid twin over temporal consistency and error accumulation. FFR struggles to capture nonlinear dynamics and APT is limited by lack of residual correction. EKF produces weakest performance as expected due to its inability to adapt to dynamic conditions. Overall, better generalizability of CL-HDT supports the correctness of architectural choices made in the proposed framework.

**Table 1:** LOTO-CV SOC metrics (21 folds).

Model	Stat	RMSE	MSE	MAE	*R^2^*	MaxAE	EndDrift
EKF	Mean	22.4685	514.6913	20.2535	0.0742	40.2407	6.6208
	Std	3.2170	142.8941	2.9116	0.3084	4.7243	7.8065
GRU	Mean	1.3424	2.0610	1.0370	0.9962	8.8150	1.4551
	Std	0.5214	1.6278	0.4611	0.0030	5.4194	0.8206
FFR	Mean	1.9237	6.6433	1.5376	0.9893	8.8721	2.7534
	Std	1.7578	15.4977	1.4626	0.0223	6.5173	4.0401
RGH	Mean	1.0356	1.3475	0.7873	0.9976	5.5715	1.2596
	Std	0.5374	1.5908	0.3916	0.0026	2.6889	1.0654
APT	Mean	2.3243	5.8995	1.7435	0.9895	5.5360	1.3892
	Std	0.7223	3.6152	0.5288	0.0069	1.7181	1.0224
CL-HDT	Mean	0.7078	0.5585	0.5763	0.9990	2.1873	0.6633
	Std	0.2458	0.3957	0.2301	0.0007	0.6078	0.5207

**Table 2:** LOTO-CV SOE metrics (21 folds).

Model	Stat	RMSE	MSE	MAE	*R^2^*	MaxAE	EndDrift
EKF	Mean	21.2574	462.2034	19.2368	0.1286	37.6179	5.8498
	Std	3.2925	140.8909	3.0242	0.3028	4.8235	7.2935
GRU	Mean	1.3016	2.0068	1.0119	0.9963	8.6418	1.4586
	Std	0.5730	1.8954	0.5359	0.0032	5.2264	0.7309
FFR	Mean	1.8678	6.1460	1.5220	0.9898	8.4010	2.6495
	Std	1.6704	14.7018	1.4158	0.0214	6.2310	3.5481
RGH	Mean	1.0296	1.4002	0.7770	0.9975	5.3874	1.0944
	Std	0.5976	1.8988	0.4368	0.0028	2.7830	1.0408
APT	Mean	2.2562	5.7298	1.7605	0.9893	5.1895	1.5120
	Std	0.8193	4.0498	0.6445	0.0078	1.8246	1.1605
CL-HDT	Mean	0.6883	0.5371	0.5653	0.9990	2.1275	0.7027
	Std	0.2578	0.4116	0.2472	0.0006	0.6187	0.5532

### 4.3 Locked holdout performance

Locked holdout performance evaluates performances on five unseen trips following hyperparameter tuning and final training with training and validation trips. Their results for SOC and SOE are presented in Tables 3 and 4, respectively. In line with LOTO-CV results, hybrid approaches are observed to outperform classical physics approaches and purely data driven approaches. For SOC, CL-HDT attains best MAE and MaxAE and maintaining competitive performance in rest metrics against RGH. Similarly for SOE, CL-HDT attains best MaxAE and maintains comparable performance against better RGH in remaining metrics. Remaining baselines stand similar to LOTO-CV evaluations in holdout test as well. Despite marginal performance difference between proposed CL-HDT and RGH in holdout test evaluation, CL-HDT is observed to be better generalizing and more adaptable to real world driving conditions.

**Table 3:** Locked holdout SOC metrics (*N* = 5 trips).

Model	Stat	RMSE	MSE	MAE	R^2^	MaxAE	EndDrift
EKF	Mean	22.6599	527.9004	19.8695	0.0015	42.2999	6.6095
	Std	4.2470	179.4878	4.4872	0.5076	5.7373	3.7845
GRU	Mean	2.1144	6.5809	1.8201	0.9913	7.2149	2.1227
	Std	1.6241	10.0455	1.6094	0.0100	2.5125	1.7583
FFR	Mean	2.1574	5.9882	1.7661	0.9914	9.9505	2.1445
	Std	1.2912	7.4151	1.1360	0.0071	7.2226	1.5471
RGH	Mean	1.6569	4.1325	1.4244	0.9946	5.0371	1.7355
	Std	1.3168	6.4835	1.3052	0.0064	2.2373	1.3847
APT	Mean	2.2017	5.0729	1.8115	0.9913	4.9114	2.2487
	Std	0.5310	2.2791	0.5248	0.0033	0.9536	1.8421
CL-HDT	Mean	1.6166	4.3888	1.4174	0.9946	4.0202	1.9335
	Std	1.4897	7.7091	1.5272	0.0078	1.5701	1.7570

**Table 4:** Locked holdout SOE metrics (*N* = 5 trips).

Model	Stat	RMSE	MSE	MAE	*R^2^*	MaxAE	EndDrift
EKF	Mean	21.6366	484.3080	19.0297	0.0238	39.8483	5.8240
	Std	4.4953	179.7917	4.7073	0.5289	5.9108	4.0792
GRU	Mean	2.0155	6.4783	1.7832	0.9913	6.5048	2.1492
	Std	1.7378	10.8768	1.7259	0.0115	2.8766	1.2665
FFR	Mean	2.0278	5.9387	1.6909	0.9918	9.4229	2.3370
	Std	1.5112	9.1096	1.3477	0.0094	8.1480	1.5974
RGH	Mean	1.6285	4.1262	1.4325	0.9945	5.0946	1.7622
	Std	1.3575	6.8528	1.3763	0.0072	2.3352	1.2258
APT	Mean	2.1392	5.0682	1.8181	0.9910	4.6370	2.3967
	Std	0.7842	3.5982	0.7950	0.0058	1.4191	1.9915
CL-HDT	Mean	1.8117	5.4111	1.6289	0.9928	4.2517	2.2348
	Std	1.6312	9.1428	1.6732	0.0098	1.6454	1.4669

### 4.4 Tracking performance and stability analysis

Further investigation beyond aggregate performance metrics were carried out on CL-HDT through prediction trajectory and temporal error tracking. Figure 2 illustrates the APT estimate trajectory and its correction using residuals in CL-HDT on best holdout trip. While the physics model captures overall trend, it has systemic deviation from actual trajectory. Deviations particularly increase around load transitions. By learning residual, CL-HDT reduces the gap between estimates and ground truth, demonstrating the advantages of closed-loop feedback.

**Figure 2:**
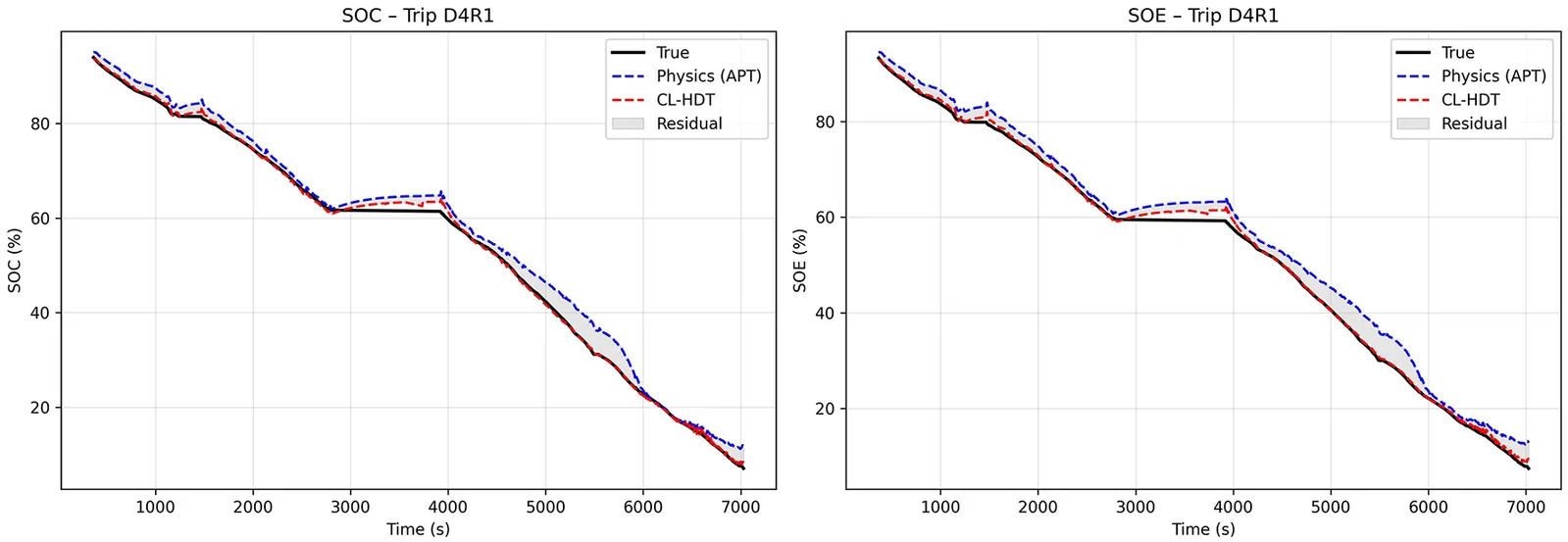
Residual correction in CL-HDT against APT for SOC and SOE estimation.

Figure 3 tracks the performance of CL-HDT in best, median, and worst-case test trips. Overall, it follows trends of both SOC and SOE. Absence of abrupt divergence and oscillations, indicate robust temporal generalizability beyond trained conditions. Even in worst case trip, error remains systematic.

**Figure 3:**
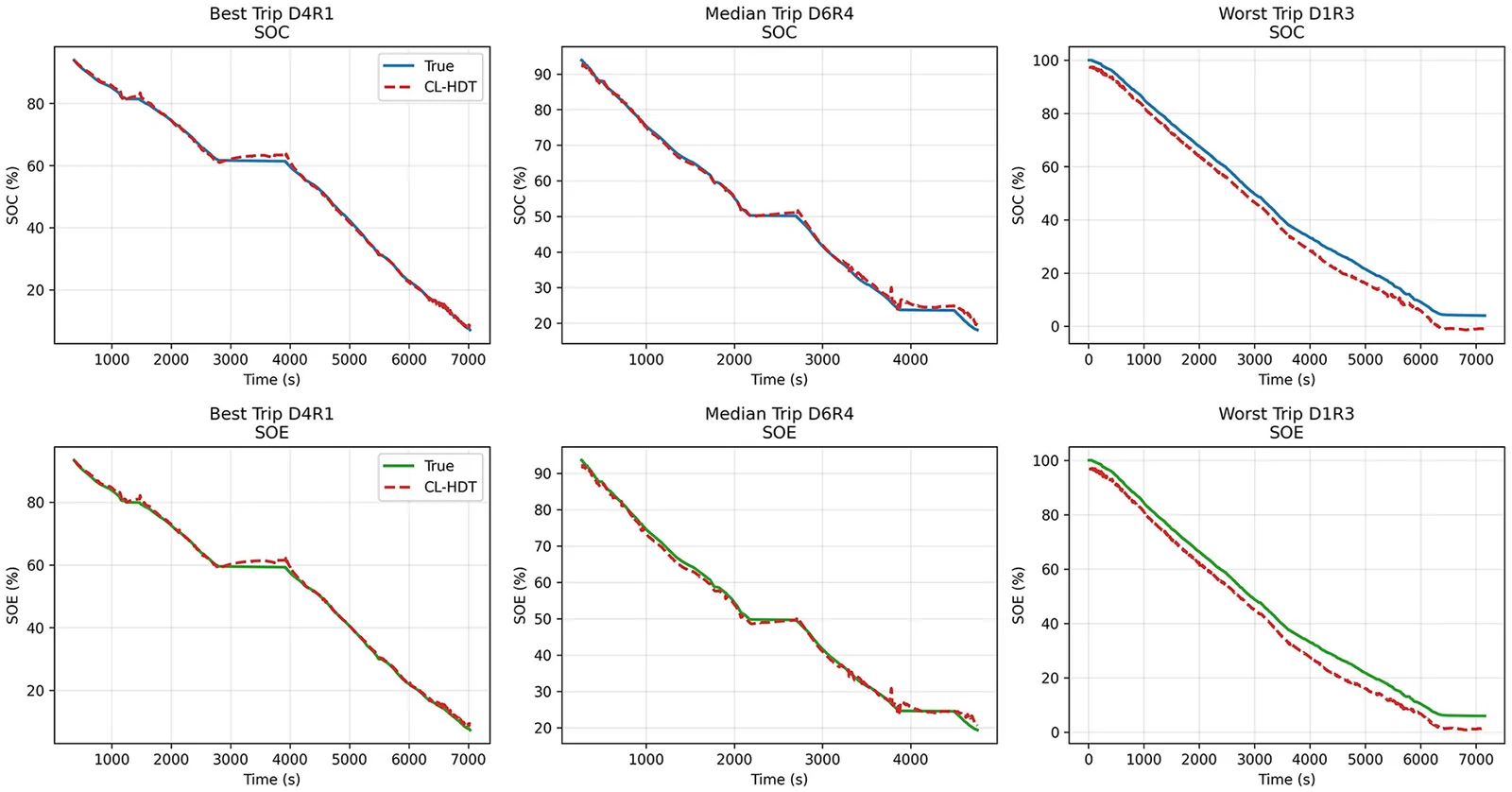
Tracking plots of best, median, and worst test trips.

Temporal stability of CL-HDT is demonstrated in Figure 4, which shows the evolution of estimation error over complete test trips. For both targets majority of error remains bounded within +/−2% without progressive increase. The observed stable temporal behavior complements results reported in previous sections to strengthen the claims of sustained prediction consistency across diverse real-world conditions.

**Figure 4:**
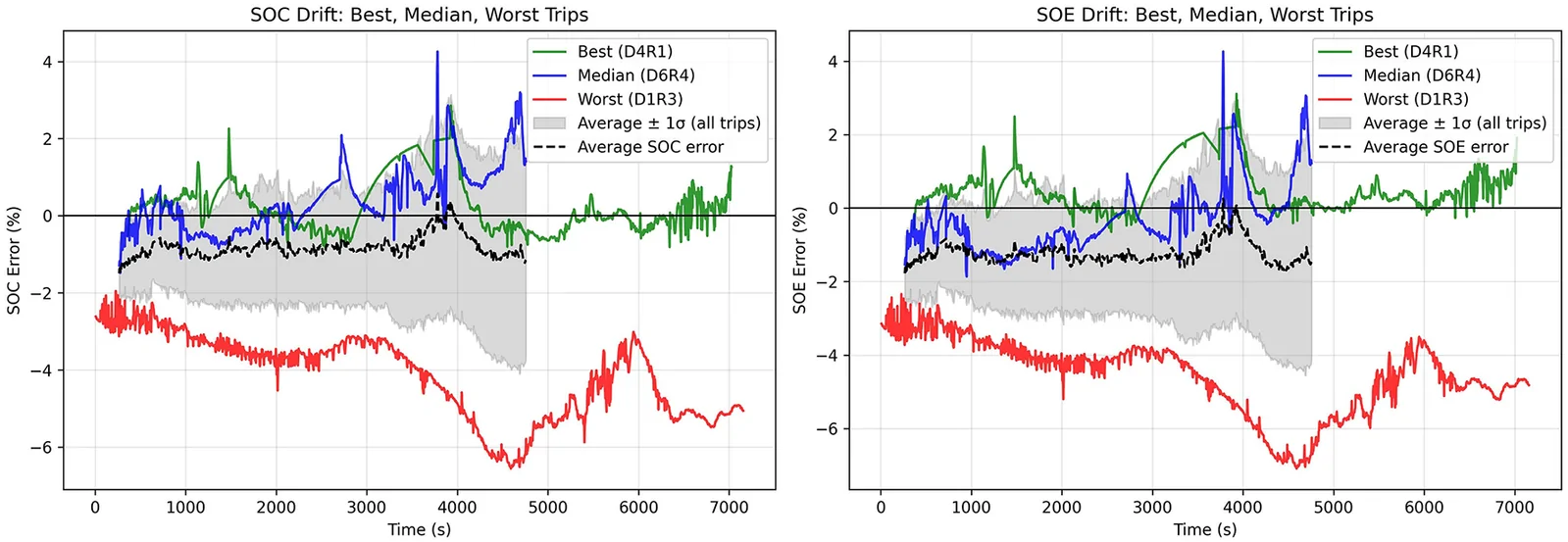
Temporal error stability of CL-HDT.

### 4.5 Component-wise ablation and error analysis

To systematically analyze the contribution of each major component of the CL-HDT, systematic ablation was carried out using locked holdout test and results are quantified in Table 5. Elimination of either recursive feedback or physics guided loss marginally degrades performance, but physics twin only configuration records significant deterioration. Although some variants marginally outperform CL-HDT in specific metrics, CL-HDT provides simultaneous balanced performance overall. These results demonstrate that complementary interaction among components of proposed CL-HDT is required to take advantage of strengths of various underlying approaches.

**Table 5:** Component-wise ablation on locked holdout test set (mean across five trips).

Variant	Output	RMSE	MAE	*R^2^*	MaxAE	EndDrift
Full CL-HDT	SOC	1.6166	1.4174	0.9946	4.0202	1.9335
	SOE	1.8117	1.6289	0.9928	4.2517	2.2348
No recursive feedback	SOC	1.7195	1.5026	0.9942	3.9804	1.8503
	SOE	1.7801	1.5678	0.9937	3.9939	2.1605
No physics loss	SOC	1.8504	1.6266	0.9931	3.8949	2.0233
	SOE	1.9265	1.7011	0.9924	4.0750	2.3192
Physics twin only	SOC	2.2454	1.8403	0.9910	5.0035	2.2544
	SOE	2.1822	1.8479	0.9907	4.7331	2.4548

Statistical behavior of prediction error is characterized in Figure 5. For both targets, distribution peaks around zero with limited positive tail demonstrating low estimation bias. Negative skew is observed due to minimal underestimation. This plot further supports the stability and robustness of the proposed CL-HDT.

**Figure 5:**
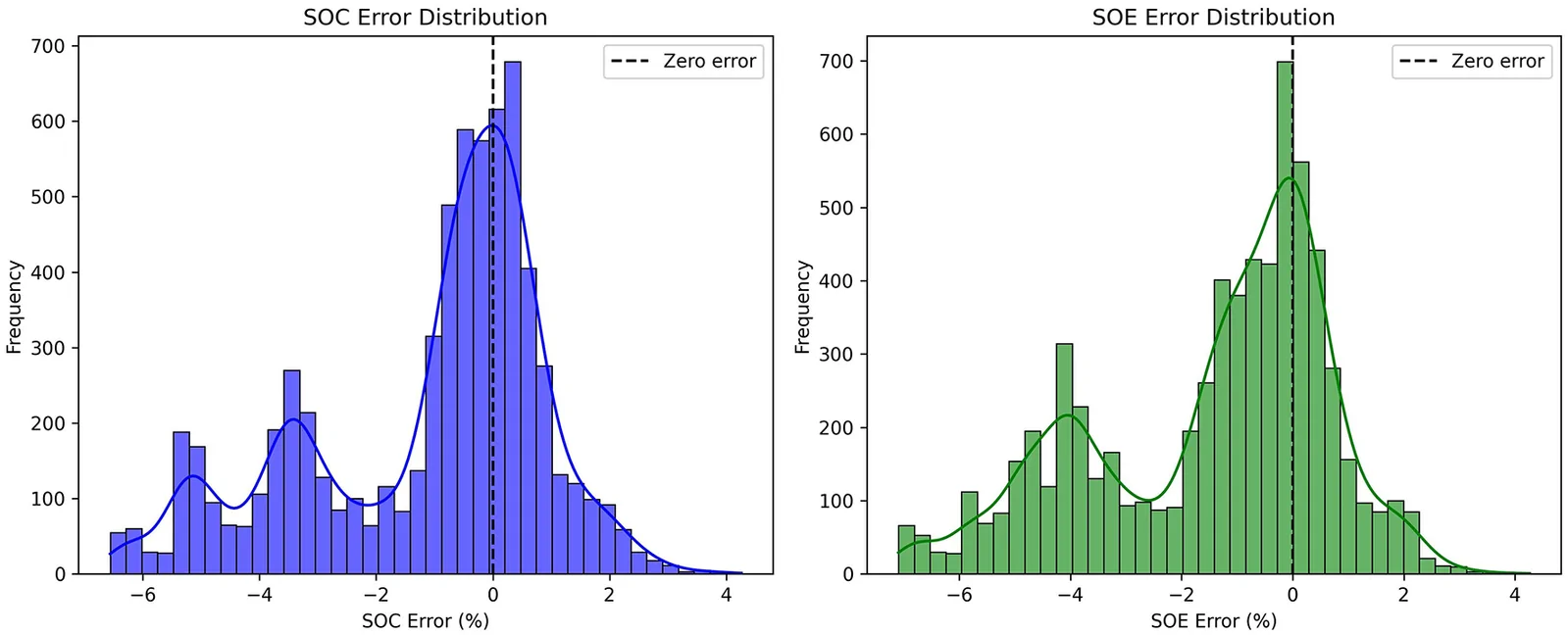
Distribution of SOC and SOE estimation errors across all holdout test set.

### 4.6 Influence of ambient operating conditions

Effect of ambient temperature and humidity on performance of CL-HDT is analyzed with locked holdout test set and depicted in Figure 6. Across conditions RMSE remains below 5%. Within limited variations of ambient conditions in test set, accuracy drops with increase in temperature, but in contrast accuracy improves with increase in humidity. Although the conditions are limited, it represents actual variation range of place of data collection and should not be considered as general trend without further validations on future datasets.

**Figure 6:**
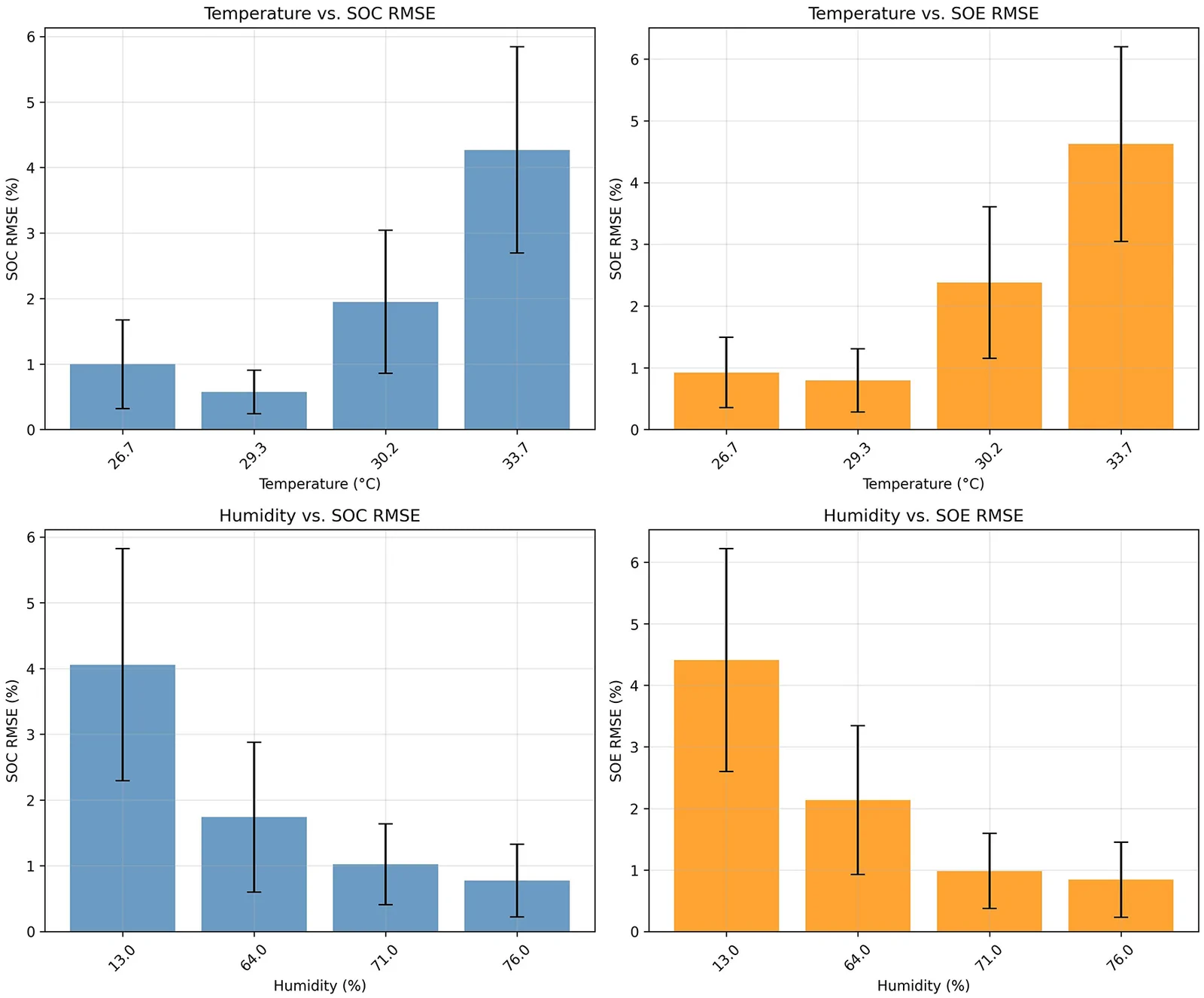
Influence of ambient conditions on SOC and SOE estimation accuracy (RMSE, mean ± standard deviation) of CL-HDT.

### 4.7 Discussion

The results demonstrate progression of performance accuracy from classical approaches to proposed CL-HDT. All the baselines and prosed architecture were processed and analyzed under identical leakage-safe pipeline to strictly observe the performance. CL-HDT reported strongest generalization due to its performance on LOTO-CV and was competitive against RGH in holdout test set. Apart from aggregate metrics, analysis like ablation, trajectory tracking, drift envelopes, error distribution, and impact of ambient conditions further highlight the temporal consistency and effective mitigation of error accumulation in CL-HDT.

Beyond CL-HDT, this work contributes by providing a rigorous evaluation framework for battery state co-estimation. Unbiased comparison of baselines act as benchmark for future works. From an application aspect, robust joint SOC-SOE estimation supports battery management system by offering reliable range prediction, charging control, power optimization, and safety under dynamic conditions. However, the findings must be interpreted within the scope of this work, which is limited to single public dataset collected from single platform with real world operating conditions. Although rigorous LOTO-CV shows generalizability, future works should experiment across battery chemistries, aging conditions, vehicle platforms, and larger datasets.

## 5 Conclusion

This work proposed Closed-Loop Hybrid Digital Twin (CL-HDT) for joint estimation of state of charge and state of energy for lithium batteries of electric cycles. CL-HDT synergizes adaptive physical modeling, recurrent residual learning, physics-guided optimization, and recursive state feedback within a unified digital twin framework. Leakage safe evaluation included leave-one-trip-out cross-validation within train set and a locked holdout test set. The proposed CL-HDT attained strongest generalization by recording LOTO-CV mean RMSE values of 0.7078% and 0.6883% for SOC and SOE respectively, while maintaining competitive performance in locked holdout test. Additional analysis like tracking, stability, residual correction, and ablation further demonstrates its temporal consistency and robustness under real diverse conditions. These findings suggest that hybrid approach with closed feedback can improve battery state estimation than traditional physics based and purely data driven approaches by overcoming their individual shortcomings.

## Data Availability

Publicly available datasets were analyzed in this study. This data can be found here: https://ieee-dataport.org/documents/real-world-dataset-soc-and-soe-estimation-electric-cycle-lithium-ion-batteries-indian.
